# Detecting and Filtering Immune-Related Adverse Events Signal Based on Text Mining and Observational Health Data Sciences and Informatics Common Data Model: Framework Development Study

**DOI:** 10.2196/17353

**Published:** 2020-06-12

**Authors:** Yue Yu, Kathryn Ruddy, Aaron Mansfield, Nansu Zong, Andrew Wen, Shintaro Tsuji, Ming Huang, Hongfang Liu, Nilay Shah, Guoqian Jiang

**Affiliations:** 1 Department of Health Sciences Research Mayo Clinic Rochester, MN United States; 2 Division of Medical Oncology Department of Oncology Mayo Clinic Rochester, MN United States

**Keywords:** immunotherapy/adverse effects, drug-related side effects and adverse reactions, pharmacovigilance, adverse drug reaction reporting systems/standards, text mining

## Abstract

**Background:**

Immune checkpoint inhibitors are associated with unique immune-related adverse events (irAEs). As most of the immune checkpoint inhibitors are new to the market, it is important to conduct studies using real-world data sources to investigate their safety profiles.

**Objective:**

The aim of the study was to develop a framework for signal detection and filtration of novel irAEs for 6 Food and Drug Administration–approved immune checkpoint inhibitors.

**Methods:**

In our framework, we first used the Food and Drug Administration’s Adverse Event Reporting System (FAERS) standardized in an Observational Health Data Sciences and Informatics (OHDSI) common data model (CDM) to collect immune checkpoint inhibitor-related event data and conducted irAE signal detection. OHDSI CDM is a standard-driven data model that focuses on transforming different databases into a common format and standardizing medical terms to a common representation. We then filtered those already known irAEs from drug labels and literature by using a customized text-mining pipeline based on clinical text analysis and knowledge extraction system with Medical Dictionary for Regulatory Activities (MedDRA) as a dictionary. Finally, we classified the irAE detection results into three different categories to discover potentially new irAE signals.

**Results:**

By our text-mining pipeline, 490 irAE terms were identified from drug labels, and 918 terms were identified from the literature. In addition, of the 94 positive signals detected using CDM-based FAERS, 53 signals (56%) were labeled signals, 10 (11%) were unlabeled published signals, and 31 (33%) were potentially new signals.

**Conclusions:**

We demonstrated that our approach is effective for irAE signal detection and filtration. Moreover, our CDM-based framework could facilitate adverse drug events detection and filtration toward the goal of next-generation pharmacovigilance that seamlessly integrates electronic health record data for improved signal detection.

## Introduction

### Background

Immunotherapy activates a patient’s immune system for therapeutic benefit against cancer [1]. One type of immunotherapy, immune checkpoint inhibition, has recently been found to be promising for the treatment of certain types of cancer. Immune checkpoint inhibitors can block negative regulators (checkpoints) of T-cell function that exist on both immune and tumor cells. This blockage could enhance antitumor immunity by allowing T cells to kill cancer cells [2]. Notably, the Nobel Prize in Physiology or Medicine in 2018 was awarded to James Allison and Tasuku Honjo for their work on immune checkpoint inhibitors [3]. From 2011 to 2017, the US Food and Drug Administration (FDA) has approved a total of 6 immune checkpoint–directed antibodies for the treatment of specific tumors. By increasing the activity of the immune system, immune checkpoint inhibitors can have inflammatory side effects, which are often termed as immune-related adverse events (irAEs) [4]. The most recognized irAEs include dermatitis, colitis, hepatitis, pancreatitis, pneumonitis, and hypophysitis [5]. IrAEs are mostly of mild to moderate severity, but at times, these can be serious, irreversible, or even fatal. Nevertheless, several studies have indicated that immune checkpoint inhibitors have a better safety profile than many traditional chemotherapies [6-8]. As these immune checkpoint inhibitor agents are new to the market, investigation of their safety profiles in real-world practice is critical [4]. Traditionally, one of the most important ways to detect postmarketing safety profiles of drugs is to conduct pharmacovigilance studies using a spontaneous reporting system (SRS) database [9]. SRS is a system whereby case reports of adverse drug events (ADEs) are voluntarily submitted by health professionals and pharmaceutical companies to the national pharmacovigilance center [10]. Several studies have focused on the irAEs post marketing pharmacovigilance through the analysis of an SRS database such as the US Food and Drug Administration’s Adverse Event Reporting System (FAERS) or World Health Organization (WHO)’s VigeBase [11-15].

Although there have been some previous studies that utilized SRS to detect irAEs, it is still essential to investigate new irAE signals to help the research community recognize a comprehensive drug safety profile for these immune checkpoint antibodies. However, it is now also recognized that traditional SRS-based ADE detection methods only focus on detecting statistically significant drug-event pairs from the SRS database, and these methods often face challenges in identifying those *new* pharmacovigilance signals automatically. Hauben and Aronson [16] proposed a widely used definition of a drug safety surveillance signal. This definition is also issued by the Council for International Organizations of Medical Sciences [17], an international organization established jointly by WHO and the United Nations Educational, Scientific and Cultural Organization, which is famous for establishing guidelines for international pharmacovigilance. According to this definition, a pharmacovigilance signal “represents an association that is new and important, or a new aspect of a known association, and has not been previously investigated and refuted.” We can note that a detected drug-event association that is not fully recognized by the previous investigation could be seen as a *signal* in this definition. These new signals can be considered to be valuable starting points for further investigation and validation. However, for a current large-scale FAERS-based pharmacovigilance study, most of the detected drug-event associations are already recognized by the existing knowledge. Some of these signals have been discovered by clinical trials before approval or by the postmarketing pharmacovigilance study [18]. To filter the known drug-event associations, health care professionals often have to manually review a substantial number of drug safety-related texts, such as drug labels or biomedical literature, to determine whether these novel ADE signals are worthy of further validation [19-21]. It is typically laborious and imprecise to manually review these ADE signals, despite the promising results achieved by existing studies, such as those by Xu et al [22,23] and Yeleswarapu et al [24]. However, these studies have focused on ranking and finding the most significant detection results and did not consider identification of novel ADE signals, which are worth further investigation. Text-mining methods allow for a more efficient way to filter the drug-event associations that are already known by existing knowledge, by extracting known ADEs from drug labels and literature. By automatically filtering out existing signals, this effort can not only discover novel irAE signals detected by the FAERS but may also reduce the labor involved in human intervention.

### Objectives

The objective of this study was to develop a framework for novel signal detection and filtration of irAEs. First, we normalized the FAERS using the Observational Health Data Sciences and Informatics (OHDSI) CDM to improve data standardization and quality to facilitate data collection and analysis. To detect irAEs, we selected all the 6 immune checkpoint inhibitors approved by the FDA before 2018 as our research object. We collected all standardized adverse event data regarding the 6 immune checkpoint inhibitors. Then, the reporting odds ratio (ROR) is utilized to detect the irAEs signal. To filter out those already known irAEs, a customized text-mining pipeline is implemented using clinical text analysis and knowledge extraction system (cTAKES) with MedDRA as a dictionary. Finally, we classified the irAE detection results into three different categories, including potentially new irAE signals.

## Methods

### Materials

#### Food and Drug Administration’s Adverse Event Reporting System

The FAERS [25] is a database that contains information on adverse event and medication error reports submitted to FDA. FAERS is designed to support postmarketing safety surveillance. All voluntary adverse event reports in FAERS could be submitted by health care professionals (such as physicians, pharmacists, nurses, and others), consumers (such as patients, family members, lawyers, and others), and manufacturers. There are 7 tables in the FAERS database, which includes patient demographic table, drug table, adverse reaction table, patient outcome table, report source table, drug therapy table, and indication table. FAERS will update quarterly and can be downloaded from the FDA website. The adverse event name in FAERS is standardized by MedDRA, a rich and highly specific standardized medical terminology. However, the drug name in FAERS is not standard, which may be a drug ingredient name, a brand name, a clinical drug component, or even a spelling error. Some other information such as drug unit and drug dosage are also nonstandard. Therefore, it is important to conduct the data preprocessing to normalize the data in FAERS before the implementation of adverse event signal detection. In this study, we used the FAERS data covering the period from September 2012 to March 2017.

#### Observational Health Data Sciences and Informatics Common Data Model

The OHDSI common data model (CDM) [26], also known as Observational Medical Outcomes Partnership CDM, is a data model designed for the systematic analysis of disparate observational databases. OHDSI CDM focuses on transforming different observational databases into a common format (data model) and a common representation (terminologies, vocabularies, coding schemes). As of February 23, 2108, version V5.3 of the CDM was released, containing 37 tables in 6 categories: standardized clinical data, standardized health system data, standardized health economics, standardized metadata, standardized vocabularies, and standardized derived elements. In fact, terminology normalization enabled by standard vocabularies with a focus on systematized nomenclature of medicine-clinical terms (SNOMED CT), logical observation identifiers names and codes, and RxNorm is a strong characteristic of the OHDSI CDM. One of the advantages of using a CDM-based database to conduct a pharmacovigilance study is that we could build a standard query as the same standard terminologies are utilized to represent the medical concepts across the different observational databases. This allows for collaborative pharmacovigilance research across different institutions.

#### Food and Drug Administration Drug Label

We searched the DailyMed website to collect the drug labels of 6 FDA-approved immune checkpoint inhibitors [27]. DailyMed, developed and maintained by the National Library of Medicine, is the official provider of FDA drug label information. We downloaded the drug labels of the 6 immune checkpoint inhibitors in January 2018. These drug labels were downloaded in the structured product labeling (SPL) format, which is a document markup standard approved by Health Level Seven (HL7) and adopted by the FDA as a mechanism for exchanging product and facility information. We extracted the text under the section WARNINGS AND PRECAUTIONS and the section ADVERSE REACTIONS from the SPL files of 6 labels as the dataset of drug label text mining.

#### Immune-Related Adverse Events–Related PubMed Literature

We retrieved literature from PubMed [28] and built an irAE-related literature text-mining dataset. The query “immune-related [All Fields] AND adverse [All Fields] AND events [All Fields]” (retrieve date: January 2018) was used to retrieve literature from PubMed. A total of 679 irAEs-related literature was obtained. Then, we downloaded the abstract of 679 papers and the full text of 20 review articles as the irAE-related literature text-mining dataset.

### Methods

**Figure 1:**
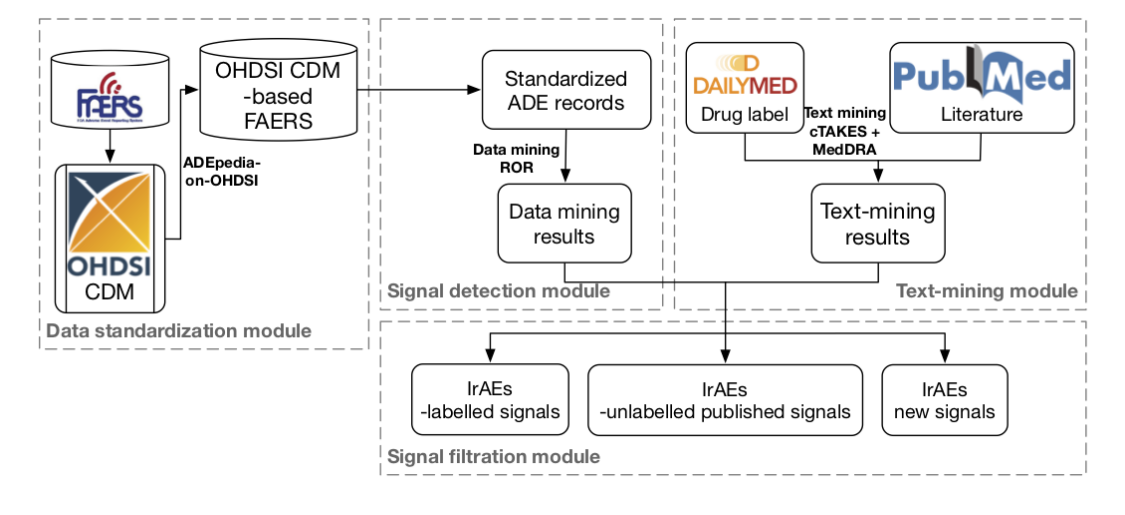
System architecture of our standards-driven framework. ADE: adverse drug events; CDM: common data model; cTAKES: clinical text analysis and knowledge extraction system; FAERS, Food and Drug Administration’s adverse event reporting system; IrAEs: immune-related adverse events; MedDRA: medical dictionary for regulatory activities; OHDSI: observational health data sciences and informatics; ROR: reporting odds ratio.

Using FAERS standardized in the OHDSI CDM, we developed a framework for signal detection and filtration of irAEs, as shown in Figure 1. The framework mainly contains 4 modules as follows (data standardization module, signal detection module, text-mining module, and signal filtration module).

#### Data Standardization Module

**Figure 2:**
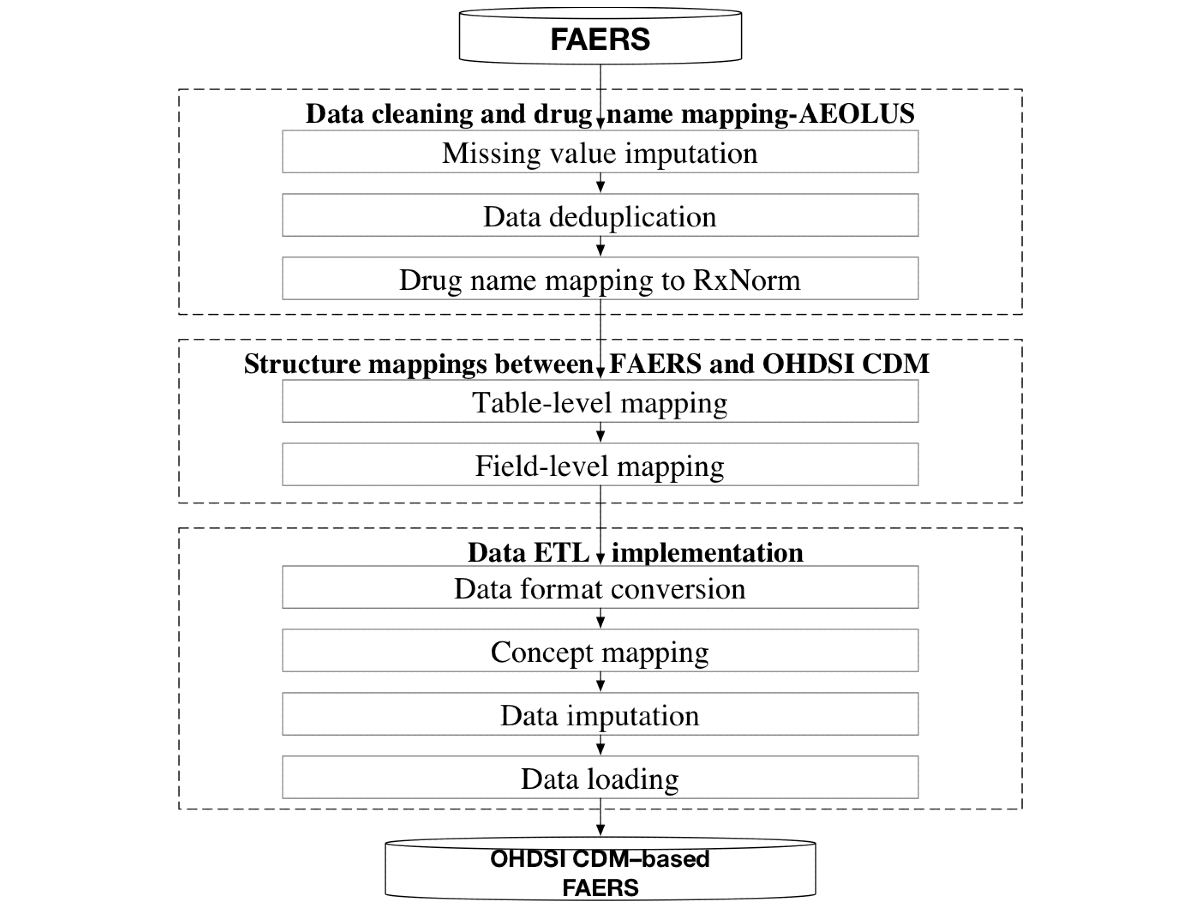
Extract, transform, and load process of converting Food and Drug Administration’s Adverse Events Reporting System into Observational Health Data Sciences and Informatics common data model. AEOLUS, adverse event open learning through universal standardization; CDM, common data model; ETL: extract, transform, and load; FAERS, Food and Drug Administration’s adverse event reporting system; OHDSI, observational health data sciences and informatics.

In FAERS, some data are nonstandard. For example, a drug name in FAERS might be a drug ingredient name, a brand name, a clinical drug component, or even a spelling error. This data standardization problem would cause inconvenience in data collection and integration and introduce bias in data analysis. In this study, we developed a next-generation pharmacovigilance signal detection platform, ADEpedia-on-OHDSI [29], to standardize FAERS and integrate it with electronic health record (EHR) data by OHDSI CDM. Specifically, we used and extended adverse event open learning through universal standardization (AEOLUS)—an integration process developed by Banda et al [30] to develop an extract, transform, and load (ETL) process to transform FAERS data into OHDSI CDM. AEOLUS focuses on building a standard process for FAERS data deduplication and tooling for mapping drug names to RxNorm concepts and outcomes to SNOMED CT concepts. We further developed an ETL tool to convert FAERS into OHDSI CDM by data structure mapping, medical concept mapping, and data imputation. The 3-step ETL process of the ADEpedia-on-OHDSI platform is shown in Figure 2. (1) Data cleaning and drug name mapping: we used AEOLUS to conduct data deduplication and drug name mapping. (2) Structure mappings between FAERS schema and OHDSI CDM schema: we created structure mappings by choosing appropriate tables or fields between the OHDSI CDM and FAERS. (3) Data ETL implementation: we designed different ETL strategies and then loaded the raw FAERS data into the OHDSI CDM. More details about the ETL process of the ADEpedia-on-OHDSI platform can be found in our published paper [29]. After the ETL process, all the standardized FAERS data were stored in the relational database in the OHDSI CDM format. In this study, we utilized pgAdmin 4 (The pgAdmin Development Team) to operate and maintain our ADEpedia-on-OHDSI platform.

#### Signal Detection Module

In this research, we define the adverse drug event (ADE) *signal* as the significant drug-adverse event associations detected by the detection algorithm using FAERS data. We implemented signal detection algorithms to detect potential irAE signals related to the 6 immune checkpoint inhibitor drugs approved by the FDA (ie, ipilimumab, pembrolizumab, nivolumab, atezolizumab, durvalumab, and avelumab). The active ingredient drug name, brand name, and the corresponding standard concept of those drugs are shown in Table 1. In addition, to facilitate the collection of irAE reports from our CDM-based FAERS, we built a standard query by checking all the synonyms of the 6 standard drug concepts in OHDSI ATHENA standardized vocabularies [31]. OHDSI concept_id related to all the ingredient/brand names is used to build SQL queries to retrieve the drug-event reports in our ADEpedia-on-OHDSI platform. The standardized SQL query for the retrieval of irAE reports is described in Multimedia Appendix 1. In addition, to validate our retrieval query, we also implemented a search by using drug ingredient/brand name verbatim texts and compared the results by different retrieval queries.

**Figure 3:**
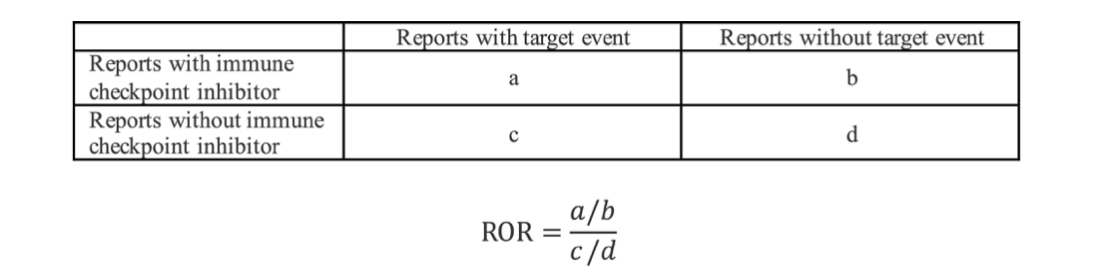
The contingency table and equation for the implementation of the reporting odds ratio.

For ADE signal detection, the ROR [32] was implemented. ROR is one of the most commonly used disproportionality statistical analysis for signal detection in SRSs such as FAERS [33]. Figure 3 illustrates the contingency table and the equation of the ROR. The ROR value and its 95% CIs were calculated to detect irAE signals. When the case report number was ≥3 and the lower limit of 95% CI of ROR was >1, the signal was considered as a positive irAE signal.

**Table 1:** The basic information of 6 immune checkpoint inhibitors.

Immune checkpoint inhibitor	Brand name	Food and Drug Administration–approved year	The Observational Health Data Sciences and Informatics concept_id (ingredient/brand name)	RxNorm concept unique identifier (ingredient/brand name)
Ipilimumab	Yervoy	2011	40238188/40238070	1094833/1094837
Pembrolizumab	Keytruda	2014	45775965/45775969	1547545/1547550
Nivolumab	Opdivo	2014	45892628/45892632	1597876/1597881
Atezolizumab	Tecentriq	2016	42629079/42629083	1792776/1792781
Durvalumab	Imfinzi	2017	1594034/1594039	1919503/1919508
Avelumab	Bavencio	2017	1593273/1593278	1875534/1875543

#### Text-Mining Module

We developed a customized text-mining pipeline to identify irAEs from the text of drug labels and irAEs-related literature using cTAKES v4.0. cTAKES is a widely used clinical information extraction tool that can discover clinically named entities and clinical events using a dictionary lookup algorithm [34]. Moreover, we implemented cTAKES using MedDRA as the dictionary to conduct text mining, so that the adverse events extracted could be standardized by MedDRA-preferred terms (PTs). Note that the result of data mining is *signal* because they have statistical significance, whereas the results extracted by text-mining pipeline are called irAE *terms*.

For drug label text mining, we collected the drug labels of the 6 FDA-approved immune checkpoint inhibitors from the DailyMed website [27]. These drug labels were downloaded in the SPL format, which is a document markup standard approved by HL7 and adopted by the FDA as a mechanism for exchanging product and facility information. Multimedia Appendix 2 shows the drug label links in DailyMed. Then, we extracted the text under the section WARNINGS AND PRECAUTIONS and the section ADVERSE REACTIONS from the SPL files of 6 labels as the dataset of drug label text mining. In addition, to evaluate the performance of cTAKES in our irAE text-mining pipeline, 2 authors (KR and GJ) manually reviewed the text under those 2 sections of the 6 drug labels and identified the irAE terms out of the drug label text, and they reached consensus via discussions. Both KR and GJ have medical backgrounds, and KR is a medical oncologist with both clinical and research expertise in treatment toxicities. The irAE terms identified from the manual review were used as a gold standard to assess the baseline performance of our text-mining pipeline, and standard measures (precision, recall, and *F*-measure) were calculated for the performance evaluation.

To identify irAEs from related literature, we searched the PubMed using the query “immune-related[All Fields] AND adverse[All Fields] AND events[All Fields].” A total of 679 irAE-related studies were found, and the abstracts were downloaded for all search results (as of January 2018). We also extracted the text from the full text of 20 review papers from the search results for text mining. The distribution of irAE-related literature by year is illustrated in Table 2, showing a trend that the number of studies on irAEs has increased significantly in recent years. We then implemented the text-mining pipeline with MedDRA as a dictionary to extract the irAE terms from both the abstracts and the full review papers of the irAE-related literature.

**Table 2:** The distribution of literature on immune-related adverse events by year (PubMed retrieve date: January 24, 2018).

Publication year	Publication number
2006	1
2007	1
2008	5
2009	7
2010	7
2011	11
2012	11
2013	37
2014	47
2015	74
2016	150
2017	260
2018	68

#### Signal Filtration Module

We reviewed all irAE signals that were identified from the signal detection and classified them into 3 categories: labeled signals (ie, those signals that could be validated by drug labels), unlabeled published signals (ie, signals that could not be found in drug labels, but in published literature), and new signals (ie, signals that could not be found either in drug labels or published literature). Then, 2 oncologists (KR and AM) manually reviewed the new signals category and gave their comments about whether an irAE signal in that category could be seen as a potentially new signal. Note that those oncologists only reviewed the detection results, and they did not have access to any other clinical data to help them ascertain what might be due to the cancer or the treatment.

## Results

### Data Standardization Results

After the ETL process, raw FAERS data were loaded into 8 OHDSI CDM tables. A total of 4,619,362 adverse event case reports were transferred into the OHDSI CDM. Among these patients, 2,577,989 (55.81%) were female, 1,603,982 (34.72%) were male, and the sex of 437,391 (9.47%) was unknown/not specified.

Table 3 shows the total numbers of irAE reports in the raw FAERS and CDM-based FAERS. It should be noted that one patient may receive more than one immune checkpoint inhibitor. We found that more irAE reports were collected after the ETL process. In CDM-based FAERS, a total of 24,595 immune checkpoint inhibitor-related AE reports were collected, compared with 24,500 in the raw FAERS before the ETL process. Of the 6 immune checkpoint inhibitors, nivolumab (Opdivo) had the most AE reports (n=12,557 before ETL and n=12,569 after ETL), followed by ipilimumab (Yervoy; n=8264 before ETL and n=8268 after ETL).

**Table 3:** Total report numbers of 6 immune checkpoint inhibitors.

Immune checkpoint inhibitor	Brand name	Adverse drug event report number (before extract, transform, and load)	Adverse drug event report number (after extract, transform, and load)
Ipilimumab	Yervoy	8264	8268
Pembrolizumab	Keytruda	5020	5099
Nivolumab	Opdivo	12,557	12,569
Atezolizumab	Tecentriq	891	893
Durvalumab	Imfinzi	27	27
Avelumab	Bavencio	5	5
Total reports	N/A^a^	24,500	24,595

^a^N/A: not applicable.

### Immune-Related Adverse Events Signal Detection Results

To provide a comprehensive perspective for irAEs, we conducted irAE signal detection at 2 different MedDRA adverse event levels: the system organ class (SOC) level and the PT level. SOC level is the highest level of MedDRA, which contains 27 groupings by etiology (eg, SOC infections and infestations), manifestation site (eg, SOC gastrointestinal disorders), and purpose (eg, SOC surgical and medical procedures). A PT term is a distinct descriptor (single medical concept) that is linked to at least one SOC. Table 4 shows the 7 positive signals detected in the SOC level.

Moreover, 94 positive signals in the PT level were detected in patients who used 1 of the 6 immune checkpoint inhibitor drugs. Among all the positive irAE signals, hypophysitis had the highest ROR value (ROR 5398.8; 95% CI 3105.1-9386.9), followed by hypopituitarism (ROR 135.1; 95% CI 106.7-171.1), blood corticotrophin decreased (ROR 59.5; 95% CI 3105.1-9386.9), adrenal insufficiency (ROR 36.1; 95% CI 31.5-41.3), and colitis (ROR 32.7; 95% CI 30.5-35.0), which means these irAEs were possibly suffered most often by the patients who were immune checkpoint inhibitors.

We also classified the irAE signals using the MedDRA SOCs to obtain a high-level understanding of the distribution of the irAE signals (shown in Table 5). Note that 1 PT might be linked to more than 1 SOC, so the total signal number in Table 5 was more than 94. All the signals we detected at the PT level could be classified into 19 SOCs. Moreover, 14 PT-level signals were categorized in *Respiratory, thoracic and mediastinal disorders*, which is the SOC with the most signals, followed by *Gastrointestinal disorders*, *Cardiac disorders*, *Infections and infestations*, and *Nervous system disorders*, of which SOCs also had more than 10 PT level signals. In addition, there was at least one PT-level signal in each of the 7 SOCs we previously detected as a positive SOC-level signal, which also validated our detection results at the SOC level. The detailed information of the 94 irAE signals is illustrated in Multimedia Appendix 3.

**Table 4:** The signal detection results at the system organ class level.

Medical Dictionary for Regulatory Activities code	System organ class	Reporting odds ratio (95% CI)
10014698	Endocrine disorders	2.98 (2.84-3.12)
10019805	Hepatobiliary disorders	2.53 (2.39-2.68)
10027433	Metabolism and nutrition disorders	1.76 (1.69-1.83)
10005329	Blood and lymphatic system disorders	1.56 (1.48-1.64)
10029104	Neoplasms benign, malignant, and unspecified (including cysts and polyps)	1.38 (1.30-1.46)
10038738	Respiratory, thoracic, and mediastinal disorders	1.27 (1.23-1.31)
10017947	Gastrointestinal disorders	1.16 (1.12-1.19)

**Table 5:** System organ class distribution of preferred term–level signals.

System organ class	Signal number
Respiratory, thoracic, and mediastinal disorders^a^	14
Gastrointestinal disorders^a^	13
Cardiac disorders	10
Infections and infestations	10
Nervous system disorders	10
General disorders and administration site conditions	9
Investigations	9
Immune system disorders	8
Endocrine disorders^a^	5
Hepatobiliary disorders^a^	5
Injury, poisoning, and procedural complications	5
Metabolism and nutrition disorders^a^	5
Skin and subcutaneous tissue disorders	5
Blood and lymphatic system disorders^a^	4
Eye disorders	4
Musculoskeletal and connective tissue disorders	4
Vascular disorders	4
Renal and urinary disorders	3
Neoplasms benign, malignant, and unspecified (including cysts and polyps)^a^	1

^a^Represents the system organ class that was detected as a positive signal in the system organ class level.

### Text-Mining Results

As mentioned previously, we utilized cTAKES with MedDRA as a dictionary to identify the irAE terms from the drug label of 6 immune checkpoint inhibitors. A total of 421 and 918 irAE terms were found by text mining of drug labels and irAEs-related literature, respectively.

Regarding drug label text mining, we found that most of the irAE terms identified by cTAKES were in the PT level of MedDRA. However, some of the irAE terms were defined as lowest-level terms (LLTs) in MedDRA. An LLT is a synonym, lexical variant, quasi-synonym, subelement, or an identical to its related PT and could be linked to only one PT. To unify the irAE terms to standard concepts at the same level, we mapped all the LLTs into PTs based on the recommendations of MedDRA and FDA. As a result, 490 irAE terms were extracted from the texts of all the 6 drug labels, comprising 474 PTs, 15 SOC, and 1 high-level term (HLT, a superordinate descriptor for the PTs linked to it). More details of the irAE terms identified from drug labels by our text-mining pipeline are provided in Multimedia Appendix 4.

For the text-mining evaluation, as mentioned in the *Methods* section, the irAE terms manually identified by 2 experts (KR and GJ) from drug labels were seen as the gold standard. Then, irAE terms extracted by the text-mining pipeline were compared with the gold standard to acquire the text-mining performance. As the text-mining pipeline, we also linked all the LLT-level irAE terms to the PT level. Using the expert-based manual review process, we identified a total of 421 distinct irAE terms from drug labels of the 6 immune checkpoint inhibitors, comprising 401 PTs, 10 SOCs, 1 HLT, and 9 terms that could not be mapped with MedDRA concepts. Multimedia Appendix 5 provides the details of the manually identified irAE terms. Table 6 shows the distribution of the irAE terms in different drug labels and the performance of our text-mining pipeline. As illustrated in the table, the overall precision, recall, and *F*-measure of our text-mining pipeline are 79.39%, 92.40%, and 85.40%, respectively, which indicates that our pipeline could provide satisfactory text-mining results and achieved the requirement of our irAE identification task.

For irAE-related literature text mining, by using our text-mining pipeline, a total of 918 unique irAE terms (in PT or higher level) were identified from 679 irAE-related abstracts and 20 irAE-related review papers, in which 306 (33.33%) terms were covered by the irAE terms that were extracted in drug labels, and the remaining 612 (66.67%) terms were not covered by the labeled irAE terms. This indicates that some unlabeled terms can be identified from our text-mining pipeline. Multimedia Appendix 6 provides the results of irAE-related literature text mining.

**Table 6:** Performance of text-mining pipeline for the identification of immune-related adverse events from drug labels of 6 immune checkpoint inhibitors.

Immune checkpoint inhibitor	Manually identified immune-related adverse events terms	Clinical Text Analysis and Knowledge Extraction System–identified immune-related adverse events terms	True positive	False positive	False negative	Precision (TP/[TP+FP]), %	Recall (TP/[TP+FN]), %	*F*-measure (2PR/[P+R]), %
Ipilimumab	122	138	103	35	19	74.6	84.4	79.2
Pembrolizumab	192	228	179	49	13	78.5	93.2	85.2
Nivolumab	215	262	202	60	13	77.1	93.9	84.7
Atezolizumab	142	157	129	28	13	82.2	90.9	86.3
Durvalumab	179	183	156	27	23	85.3	87.2	86.2
Avelumab	146	176	130	46	16	73.9	89.0	80.8
Total	421	490	389	101	32	79.4	92.4	85.4

### Signal Filtration Results

**Figure 4:**
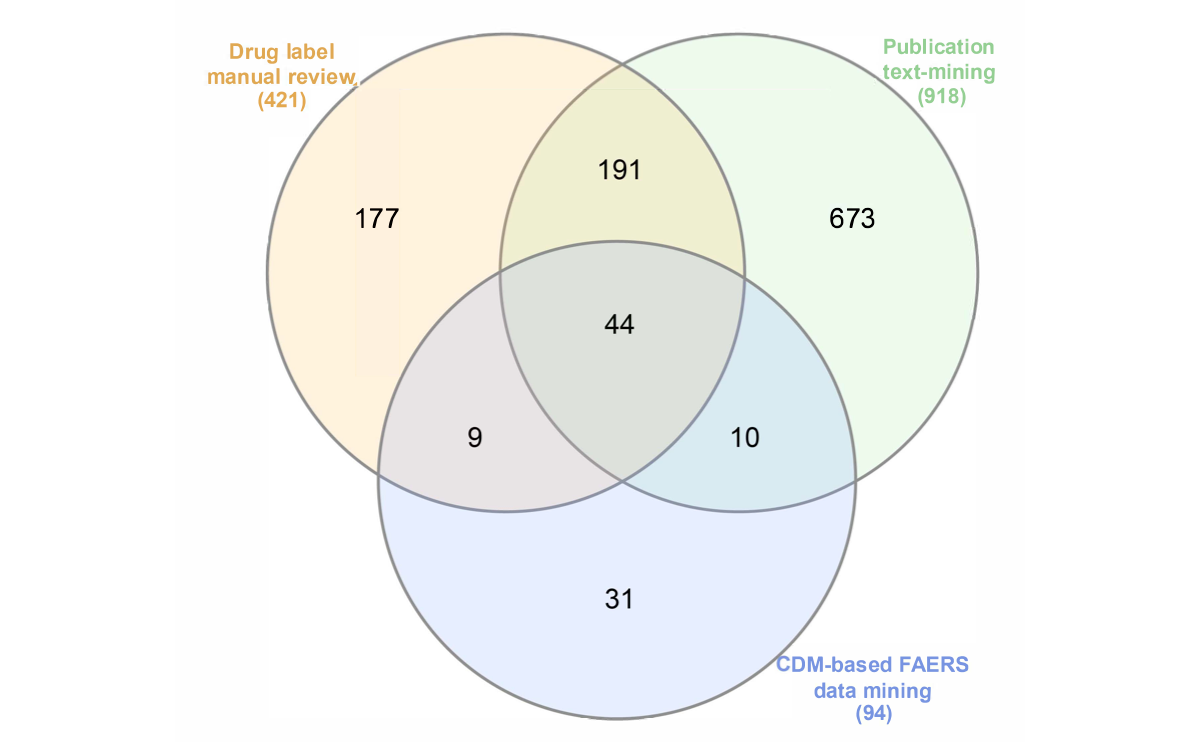
Venn diagram illustrating the immune-related adverse events terms detected from different sources. CDM, common data model; FAERS, Food and Drug Administration’s adverse event reporting system.

To filter the irAE signals we detected, we compared all 94 irAE signals with the text-mining results and then classified all the signals into 3 categories as per our definition in the *Methods* section. Figure 4 shows the overlap of the irAEs terms identified in 3 different mining tasks. In total, 1135 unique irAE terms were identified by CDM-based FAERS data mining, drug label manual review, and irAE-related literature text mining. Out of 94 positive signals in the PT level detected using CDM-based FAERS, 53 signals (56%) were the labeled signals we identified from drug labels, 10 signals (11%) were the unlabeled published signals identified from the literature, and 31 signals (33%) were potentially new signals that were not covered by drug labels and literature (as shown in Table 7). Multimedia Appendix 7 demonstrates the details of labeled signals, unlabeled published signals, and new signals. For a further manual review, 2 oncologists separately marked 15 and 8 signals that were *possibly new*, after reviewing a total of 31 irAE signals in the new signal category. The kappa coefficient value of the review is 0.48, which showed a *moderate agreement* between the 2 oncologists [35]. Moreover, 7 irAE signals were identified as potentially new signals by both the oncologists (as shown in Table 7).

**Table 7:** A list of 31 potentially new signals not identified in drug labels or literature (ranked by reporting odds ratio).

Medical Dictionary for Regulatory Activities code	Preferred term	System organ class	Reporting odds ratio (95% CI)
10005452	Blood corticotrophin decreased	Investigations	59.49 (34.44-102.74)
10053481	Bronchopleural fistula^a^	Respiratory, thoracic, and mediastinal disorders	19.51 (6.96-54.67)
10006437	Bronchial fistula^a^	Respiratory, thoracic, and mediastinal disorders	19.01 (6.79-53.20)
10042569	Superior vena cava syndrome	Vascular disorders/ neoplasms benign, malignant, and unspecified (including cysts and polyps)	10.62 (5.78-19.51)
10061457	Facial nerve disorder^a^	Nervous system disorders	9.51 (3.48-25.97)
10044291	Tracheal obstruction^a^	Respiratory, thoracic, and mediastinal disorders/ injury, poisoning, and procedural complications	7.83 (2.47-24.87)
10058838	Enterocolitis infectious	Gastrointestinal disorders/infections and infestations	7.64 (3.77-15.51)
10065764	Mucosal infection	General disorders and administration site conditions/infections and infestations	7.13 (2.25-22.59)
10013832	Duodenal perforation	Gastrointestinal disorders	6.50 (2.88-14.68)
10006440	Bronchial obstruction^a^	Respiratory, thoracic, and mediastinal disorders	6.34 (3.13-12.82)
10061145	Eyelid function disorder^a^	Eye disorders	5.73 (1.82-18.09)
10007196	Capillary leak syndrome^a^	General disorders and administration site conditions/vascular disorders	5.62 (2.78-11.35)
10010276	Conduction disorder	Cardiac disorders	4.92 (2.19-11.07)
10036774	Proctitis	Gastrointestinal disorders	4.90 (2.82-8.50)
10021305	Ileal perforation	Gastrointestinal disorders	4.09 (1.30-12.84)
10009995	Colonic fistula	Gastrointestinal disorders	3.81 (1.21-11.95)
10064774	Infusion site extravasation	Injury, poisoning, and procedural complications/general disorders and administration site conditions	3.51 (2.30-5.36)
10051341	Bile duct stenosis	Hepatobiliary disorders	3.45 (1.42-8.35)
10042241	Stridor	Respiratory, thoracic, and mediastinal disorders	3.15 (1.41-7.06)
10035623	Pleuritic pain	Respiratory, thoracic, and mediastinal disorders	3.12 (1.67-5.82)
10025256	Lymphocyte count decreased	Investigations	2.97 (2.29-3.85)
10063057	Cystitis noninfective	Renal and urinary disorders	2.83 (1.05-7.60)
10005630	Blood lactate dehydrogenase increased	Investigations	2.81 (2.10-3.76)
10041549	Spinal cord compression	Nervous system disorders	2.81 (1.66-4.76)
10008612	Cholecystitis	Hepatobiliary disorders	2.59 (1.87-3.58)
10041103	Small intestinal perforation	Gastrointestinal disorders	2.46 (1.02-5.94)
10003662	Atrial flutter	Cardiac disorders	2.45 (1.50-4.02)
10036206	Portal vein thrombosis^a^	Vascular disorders/hepatobiliary disorders	2.43 (1.30-4.53)
10029164	Nephrotic syndrome	Renal and urinary disorders	2.37 (1.42-3.94)
10003673	Atrioventricular block complete	Cardiac disorders	1.85 (1.09-3.13)
10003504	Aspiration	Respiratory, thoracic, and mediastinal disorders	1.60 (1.02-2.51)

^a^Identified as potentially new signals by both oncologist reviewers.

## Discussion

### Principal Findings

To the best of our knowledge, this is the first comprehensive, novel signal detection and filtration study of irAEs utilizing multiple drug safety data sources. We proposed a framework to detect the irAE signals from a standardized FAERS database and utilized a text-mining pipeline with drug labels and existing literature to discover *potentially new irAE signals*. Our framework could facilitate ADE detection and filtration toward the goal of next-generation pharmacovigilance. This could decrease the labor consumption in new irAE signal selection and provide stronger hypotheses for further experimental validation. In the future, the results of this work will be potentially combined with the EHR data to leverage the real-world discovery of treatment toxicities.

We utilized standard OHDSI CDM to represent the FAERS data (ie, the ADEpedia-on-OHDSI platform) and created standard queries for signal detection, which provides a solid data infrastructure to make the queries portable and signal detection results reproducible. More importantly, through the comparison of data collection between the raw FAERS and CDM-based FAERS, we found that the OHDSI CDM could improve the precision of the data collection. For example, for the drug pembrolizumab, we collected 5099 reports from the CDM-based FAERS, 79 reports more than those we collected from the raw FAERS. To illustrate the reason for the difference in data collection using the OHDSI CDM-based FAERS, we manually checked the data we collected from the raw FAERS and CDM-based FAERS. For example, we discovered that when we utilized the standard OHDSI concept id as a query to retrieve CDM-based FAERS, we could collect more reports regarding the drug name “MK-3475,” which was the original name of pembrolizumab in its early development, in addition to those reports we retrieved when we used the drug ingredient name “Pembrolizumab” and brand name “Keytruda.” This meant that we improved the true positive rate and precision for data collection. Moreover, we could save time for collecting data through a standard query. For example, for pembrolizumab, it took 9.4 seconds to pull all data from CDM-based FAERS with our standard query, in contrast to approximately 70 seconds for the raw FAERS data collection through a fuzzy search query with the drug/brand name terms.

We also leveraged text-mining technology to process unstructured drug safety data. We implemented our text-mining pipeline on the drug label and irAE literature with MedDRA as a dictionary to identify the irAE terms. In addition, to evaluate the performance of our text-mining pipeline, the irAEs in drug labels were manually reviewed and extracted as a gold standard. As a result, the overall precision, recall, and *F*-measure of all 6 drug labels were 79.39%, 92.40%, and 85.40%, respectively. These results indicate that although there were some false positive terms (about 20%) found in our text-mining results, most irAE terms (92.40%) in the text could be extracted correctly by our pipeline. Moreover, we checked the underlying reasons behind the false positive terms. We found that most of these terms are related to the laboratory test name, such as *Alanine aminotransferase*, *Blood alkaline phosphatase*, and so on. Actually, for laboratory tests, the appropriate terms matched with irAEs should be the specific abnormal test result terms, such as *Alanine aminotransferase increased* and *Blood alkaline phosphatase increased*, which were also found in both gold standard and text-mining results. Given this analysis, we plan to improve the precision of our text-mining pipeline using a rule-based approach in a future update.

### Limitations and Future Work

Our framework provides an automatic process to detect novel irAE signals that are more valuable for implementing further experimental validation. It also profoundly saves the experts’ time in reviewing drug labels and the literature to filter the known ADEs. In total, we detected 94 irAE signals from FAERS. After the filtering, 31 irAEs were classified into the *new irAE signals* category. In addition, 7 out of 31 signals in the *new signal* category were identified as *potentially new* irAE signals by both the oncologists, which indicated that some of the new signals detected by these algorithms might be false positive. According to the oncologists’ review, some of the signals were marked as *not new*. We consider that one of the main reasons for the false positive signal was that sometimes the description of irAEs by the MedDRA PTs was not so accurate. For example, some detected new signals might be a hyponym of a known irAE, that is, they are more specific than a general irAE. For example, *Conduction disorder* and *Atrioventricular block complete* were detected as new irAE signals by our pipeline. However, the oncologist reviewers judged that these are not new because they are types of arrhythmias, which belong to cardiotoxicity and are known to be associated with the immune checkpoint blockade [36]. Moreover, 2 of the potentially new signals, *bronchopleural fistula/bronchial fistula* and *tracheal obstruction/bronchial obstruction*, are almost the same medical concept. Thus, there is a need to develop a harmonized terminology to report and describe irAEs to interpret safety data more accurately in monitoring missions. One of our previous studies discussed the possibility of leveraging the Common Terminology Criteria for Adverse Events (CTCAE) for irAE standardization. We found that the CTCAE needs an extension to meet the irAE standardization task [37]. Similarly, other studies have also demonstrated how to build a terminology to standardize irAEs [38,39]. In future work, we will improve the text-mining process to facilitate the development of ADE terminology. First, to create a harmonized irAE terminology and make it more suitable for detecting irAE signals from other data sources such as EHR, we will extend the text-mining dictionary to SNOMED CT. Second, we will further improve the performance and automation of our text-mining pipeline to make the terminology easier to update and maintain. Third, we will further evaluate the text-mining pipeline to investigate its feasibility of developing specific terminology sets for other ADE categories.

For those irAE signals in the *new signal* category, some of them were marked as *possibly new* by 2 oncologists because these adverse events may be induced by or associated with cancer, the complication of surgery or radiation, other drugs administered in the cancer treatment regimen, or drug- drug interactions. For example, *Bronchopleural fistula*, *bronchial obstruction*, and *bronchial fistula* all can occur due to a pulmonary cancer or as a complication of pulmonary surgery or radiation [40]. However, FAERS does not provide information such as timeline details about the drug administration/diagnosis/event, which is an obstacle to confirming whether a signal is caused by the treatment or other conditions. Therefore, expert reviews from oncologists are important for our detection pipeline to control false positive signal results. Moreover, as mentioned in the *Methods* section, oncologists also need more clinical data to further validate the relationship between these irAE signals and immunotherapy drugs. Longitudinal observational databases such as EHRs have increasingly been used for further evaluation of adverse event signals. Compared with FAERS data, EHRs not only contain information about patients who suffer ADEs but also provide a more complete medical history of the patients, including treatments, conditions, and potential risk factors. Accordingly, EHRs could be an additional data source for irAE signal detection [41]. We are actively working on integrating EHR data with our ADEpedia-on-OHDSI platform, which can scale to support more advanced signal detection [42]. Our standard-driven platform integrates FAERS data and EHR data together by using the same data standards that could facilitate pharmacovigilance research based on real-world data. Our platform can not only improve data quality but can also facilitate the data collection for comprehensive ADE detection or cross-validation. In the future, we will try to conduct more comprehensive ADE detection studies based on real-world data to overcome the false positive issue. Furthermore, we will also consider utilizing semantic web technology to develop more ADE mining methods.

### Conclusions

In this study, we developed and evaluated a novel standards-based framework for signal detection and filtration of irAEs using both the OHDSI CDM and text-mining technologies. We demonstrated that our approach is effective for novel irAE signal detection and filtration; meanwhile, the CDM-based platform provides an infrastructure that would enable the seamless integration of EHR data for improving signal detection in the future.

## Appendix

Multimedia Appendix 1 The standardized SQL query for the irAE record retrieving.

Multimedia Appendix 2 The drug label links of six FDA-approved mAb drugs in DailyMed.

Multimedia Appendix 3 The detail information of 94 irAE signals at PT level.

Multimedia Appendix 4 The irAE terms identified form drug labels by the text-mining pipeline.

Multimedia Appendix 5 The detail of the manually identified irAE terms from the drug labels.

Multimedia Appendix 6 The irAE terms identified form irAE-related literature by the text-mining pipeline.

Multimedia Appendix 7 The detail of labeled irAE signals, unlabeled published irAE signals, and new irAE signals.

## Abbreviations

ADE: adverse drug event
AEOLUS: adverse event open learning through universal standardization
CDM: common data model
cTAKES: clinical text analysis and knowledge extraction system
CTCAE: common Terminology Criteria for Adverse Events
EHR: electronic health record
ETL: extract, transform, and load
FAERS: Food and Drug Administration’s Adverse Event Reporting System
FDA: Food and Drug Administration
HL7: Health Level Seven
HLT: high-level term
irAEs: immune-related adverse events
LLTs: lowest-level terms
MedDRA: Medical Dictionary for Regulatory Activities
OHDSI: Observational Health Data Sciences and Informatics
PT: preferred term
ROR: reporting odds ratio
SNOMED CT: systematized nomenclature of medicine-clinical terms
SOC: system organ class
SPL: structured product labeling
SQL: Structured Query Language
SRS: spontaneous reporting system
WHO: World Health Organization
