# Supplementary material for: Detecting and Filtering Immune-Related Adverse Events Signal Based on Text Mining and Observational Health Data Sciences and Informatics Common Data Model: Framework Development Study
Source: JMIR Med Inform. 2020 Jun 12;8(6):e17353. doi: 10.2196/17353 (PMC7320306; doi:10.2196/17353)
Supplement: Multimedia Appendix 1 [file medinform_v8i6e17353_app1.docx]

S1.The standardized SQL query for the irAE record retrieving:

select * from public.drug_exposure a, public.observation b

where a.person_id = b.person_id

and (a.drug_concept_id = '40238188' or a.drug_concept_id = '40238070'

or a.drug_concept_id = '45775965' or a.drug_concept_id = '45775969'

or a.drug_concept_id = '45892628' or a.drug_concept_id = '45892632'

or a.drug_concept_id = '42629079' or a.drug_concept_id = '42629083'

or a.drug_concept_id = '1594034' or a.drug_concept_id = '1594039'

or a.drug_concept_id = ' 1593273' or a.drug_concept_id = '1593278')
