# Supplementary material for: Detecting and Filtering Immune-Related Adverse Events Signal Based on Text Mining and Observational Health Data Sciences and Informatics Common Data Model: Framework Development Study
Source: JMIR Med Inform. 2020 Jun 12;8(6):e17353. doi: 10.2196/17353 (PMC7320306; doi:10.2196/17353)
Supplement: Multimedia Appendix 2 [file medinform_v8i6e17353_app2.docx]

Supplemental File 2. The drug label links of six FDA-approved mAb drugs in DailyMed

| Drug Name (mAb) | Drug Label Link in DailyMed |
| --- | --- |
| YERVOY- ipilimumab | https://dailymed.nlm.nih.gov/dailymed/drugInfo.cfm?setid=2265ef30-253e-11df-8a39-0800200c9a66 |
| KEYTRUDA- pembrolizumab | https://dailymed.nlm.nih.gov/dailymed/drugInfo.cfm?setid=9333c79b-d487-4538-a9f0-71b91a02b287 |
| OPDIVO- nivolumab | https://dailymed.nlm.nih.gov/dailymed/drugInfo.cfm?setid=f570b9c4-6846-4de2-abfa-4d0a4ae4e394 |
| TECENTRIQ- atezolizumab | https://dailymed.nlm.nih.gov/dailymed/drugInfo.cfm?setid=6fa682c9-a312-4932-9831-f286908660ee |
| IMFINZI- durvalumab | https://dailymed.nlm.nih.gov/dailymed/drugInfo.cfm?setid=8baba4ea-2855-42fa-9bd9-5a7548d4cec3 |
| BAVENCIO- avelumab | https://dailymed.nlm.nih.gov/dailymed/drugInfo.cfm?setid=5cd725a1-2fa4-408a-a651-57a7b84b2118 |
